# The human resource implications of improving financial risk protection for mothers and newborns in Zimbabwe

**DOI:** 10.1186/1472-6963-13-197

**Published:** 2013-05-28

**Authors:** Yotamu Chirwa, Sophie Witter, Malvern Munjoma, Wilson Mashange, Tim Ensor, Barbara McPake, Shungu Munyati

**Affiliations:** 1 Biomedical Research and Training Institute, Harare, Zimbabwe; 2 IIHD, Queen Margaret University, Edinburgh, Scotland, UK; 3 Leeds Institute of Health Sciences, University of Leeds, Leeds, UK

**Keywords:** Human resources for health, Zimbabwe, Reproductive, Maternal and newborn health, Financial access, User fees

## Abstract

**Background:**

A paradigm shift in global health policy on user fees has been evident in the last decade with a growing consensus that user fees undermine equitable access to essential health care in many low and middle income countries. Changes to fees have major implications for human resources for health (HRH), though the linkages are rarely explicitly examined. This study aimed to examine the inter-linkages in Zimbabwe in order to generate lessons for HRH and fee policies, with particular respect to reproductive, maternal and newborn health (RMNH).

**Methods:**

The study used secondary data and small-scale qualitative fieldwork (key informant interview and focus group discussions) at national level and in one district in 2011.

**Results:**

The past decades have seen a shift in the burden of payments onto households. Implementation of the complex rules on exemptions is patchy and confused. RMNH services are seen as hard for families to afford, even in the absence of complications. Human resources are constrained in managing current demand and any growth in demand by high external and internal migration, and low remuneration, amongst other factors. We find that nurses and midwives are evenly distributed across the country (at least in the public sector), though doctors are not. This means that for four provinces, there are not enough doctors to provide more complex care, and only three provinces could provide cover in the event of all deliveries taking place in facilities.

**Conclusions:**

This analysis suggests that there is a strong case for reducing the financial burden on clients of RMNH services and also a pressing need to improve the terms and conditions of key health staff. Numbers need to grow, and distribution is also a challenge, suggesting the need for differentiated policies in relation to rural areas, especially for doctors and specialists. The management of user fees should also be reviewed, particularly for non-Ministry facilities, which do not retain their revenues, and receive limited investment in return from the municipalities and district councils. Overall public investment in health needs to grow.

## Background

A paradigm shift in global health policy regarding user fees has been evident in the last decade with a growing consensus that user fees are regressive and undermine equitable access to essential health services [1]. In particular, a concern that pregnant women and children under five are negatively affected by such financial barriers has prompted many low and middle income countries to reconsider levying user charges by ensuring either more thorough implementation of exemption or waiver mechanisms, significant reduction in fee levels or their abolition altogether [2,3]. Such a policy shift has consequences for the health system across a number of dimensions, including the search for replacement revenue and ensuring quality in responding to the changes in utilization [1,4]. Both of these anticipated consequences raise specific concerns for human resources for health (HRH), yet this issue has been frequently overlooked until recently.

This article examines the case of Zimbabwe, which faces challenges in offering access to health services to its population, and also in retaining a skilled and well-distributed health workforce, after more than a decade of political conflict and economic collapse. Using existing data and studies, linked with small-scale qualitative fieldwork, this article examines current access to care, especially financial access, focussing on reproductive, maternal and newborn health services, and also the challenges facing HRH in Zimbabwe. The scope for increasing demand for services is investigated, and the implications for staffing analysed. This contributes to a wider study aiming to understand the implications of changing user fee regimes for human resources for health.

## Methods

The research is based on a mix of research methods which included secondary data and interviews with health officials. According to the Biomedical Research and Training Institute-Institutional Review Board, the study meets their criteria for exemption from ethical review.

### Literature and policy analysis

A thorough review of literature was undertaken on the themes of health financing policy, user fees, reproductive health and human resources for health in Zimbabwe. The literature review included searching peer-reviewed and grey literature in recognized electronic databases and websites. Sources for the literature included the Ministry of Health & Child Welfare (MoHCW), Department for International Development UK (DFID), United States Agency for International Development (USAID), World Health Organisation (WHO), health research institutions, mission umbrella organisations, the Centre for Disease Control (CDC) and professional associations. Key policy documents relating to user fees and HRH were also obtained and analysed.

### Secondary data analysis

National data sets relating to staffing, staff remuneration and conditions, utilisation of services and other relevant indicators such as poverty and income levels were sought and analysed. Sources for these included the National Health Information System (NHIS), the database held by the Department of human resources (HR) in the MoHCW, and nationally published statistics produced by Zimbabwe National Statistics Agency (ZIMSTAT). Data from these sources were retrieved into an Excel spreadsheet, disaggregated to the lowest level permitted by the data.

### Key informant interviews

Key informant interviews (KII) were carried out with a selected number of experts and practitioners in Harare and one field site. The research district was selected because of its proximity to Harare and the fact that it contains a variety of communities, including mining and farming and new resettlement areas.

A semi-structured questionnaire was drawn up for the KII. It covered the following topics: current policies on user fees; current policies on exemptions; plans to reform user fees; major constraints facing HRH; the interaction of fees and HRH; and any evidence on the impact of fees and the recent dollarisation on both users and staff. (Dollarisation was the shift from using the Zimbabwe dollar, whose value had collapsed due to hyper-inflation, to the US dollar as national currency in April 2009).

The selection of the experts and practitioners was purposive. 13 individuals were interviewed – two at national level, with responsibility for human resources, and 11 at district level, in the district health office, the district hospital and health centres.

### Focus group discussions

Focus Group Discussions (FGDs) were held in three different types of area in the research district:

1. Low income urban area

2. Communal area

3. New resettlement farming/resettlement community

The key target groups were users of RMNH services and service providers. We conducted five FGDs as follows:

1. Nurse trainees (based at the district hospital) (one group)

2. User groups consisting of women at the community level (identified through the Village Health Workers) (three groups – one per area)

3. One FGD with traditional birth attendants in a rural area

The total number of participants was 43 (all female, with one exception).

Topic guides were drawn up for the two groups. The topic guide for trainee nurse midwives asked about motivation for joining the profession; their views on user fees; and factors encouraging their retention and performance. The topic guide for the community level focussed on health seeking behaviour and views on quality of care; current payments for RMNH services and how affordable they are; and users’ views on fees.

### Data analysis

Quantitative data were collated and analysed using Excel. The WHO tool produced by the Making Pregnancy Safer (MPS) department was also used to project staffing needs and gaps.

Qualitative data from the field were transcribed and analysed through categorisation of themes and content analysis. This was compared with findings from the literature review and policy analysis, as well as the secondary data, to reach overall conclusions and recommendations.

## Results

### Background on health system since independence

Zimbabwe inherited the Rhodesian health care system at independence in 1980. Health services were divided along racial lines and distribution of available resources were highly skewed towards hospital service provision for the small white population at the expense of the indigenous population. There were profound imbalances in the allocation of physical, financial and human resources in the health sector before independence [5-10].

After independence, the Government expressed the intention to focus its efforts on redressing the existing inequalities by investing especially in health services in rural areas. The government viewed health as an integral part of development and as a human right [11] and this guided the post-independence government’s health policy, resource allocation decisions and human resource development [12]. By 1989 the number of rural health centres and clinics had increased from 247 at independence to 1062 [9] resulting in much better geographical accessibility of primary care services. In the 1980s and early 1990s these health centres were adequately manned with a doctor to patient ratio of on average 1: 6000 in public institutions [13].

Hit by low growth and an Economic and Social Adjustment Plan in the 1990s, the focus shifted from equity to cost recovery and greater efficiency. While the second *Health for all Action Plan* for the period 1991–95 maintained the focus of its predecessor on equity, the plan put more emphasis on quality of care, effective use of resources, value for money and appropriateness of services, including increased use of private sector facilities [14]. This was accompanied by changes in the policy on user fees and HRH.

The World Bank [15] estimated that the gross national income (GNI) per head declined by 54% between 2000 and 2005. The estimated gross domestic product (GDP) per capita of US$268 (at purchasing power parity) placed Zimbabwe as one of the poorest countries in the world. A declining national income, a huge national debt, recurrent droughts, widespread Human Immunodeficiency Virus (HIV) and Acquired Immune Deficiency Syndrome (AIDS) all contributed to a weakening health system since 1990.

Whilst the 1980s showed a general improvement in most of the major health indicators and service utilization, attributable to the expansion and improvements in the area of primary health care [10,16,17], signs of deterioration were evident in the 1990s. The trends were a reversal of the gains made in the previous decade [12]. Between 1990 and 2008, life expectancy at birth fell from 62 to 44 years [18]. Under-five mortality (U5MR) and infant mortality rate (IMR) rose from 77 and 53 per 1,000 live births in 1992 to 94 and 67 in 2009. Maternal mortality increased dramatically from 390 per 100,000 births in 1990 to 790 in 2008 [19]. Skilled attendance at delivery dropped from 73% in 1999 to 60% in 2009 (Global Observatory, which notes that the level of skill is not specified). Neonatal mortality rose and then fell over the period of 1990–2009, ending at the same level that it started (27 per 1,000 births) [19].

The health system is dominated by the public sector, which provides an estimated 65% of health care services in the country, although in rural areas the mission sector plays a major role [20]. The private for profit sector is focussed in urban areas. Some facilities are operated by municipalities, which receive block grants from government. The Access to Health Care Services Study of 2007 found that most communities live within a 5 km radius from their nearest health facilities, whilst 23% live between 5 to 10 km and 17% are over 10 km from their nearest health centre [21].

Assessing trends in health financing is complicated by the years of hyperinflation^a^. However, public health spending was reported to constitute less than one percent of GDP (an estimated 0.02%) in 2009. Per capita public spending was reported to be $5.77 in 2009 [22]. This compares to an average of $12 for governments in low-income African countries [23].

## Financial access to reproductive, maternal and neonatal health services

### History of user fees in Zimbabwe

User fees existed in Zimbabwe from independence. Those with incomes of less than Zimbabwe Dollar (ZWD) 150 per month qualified for free health care, however this covered a decreasing proportion of the population as inflation reduced the real value of the threshold [24]. In 1991, cost recovery measures were further strengthened at the behest of the World Bank [12]. The table below shows some of the main changes in user fees policy in the 1990s (Table 1).

**Table 1:** Changes in user fee policy during the 1990s, Zimbabwe

	
Early 1991	Enforcement of user fee collection at all health facilities at the start of ESAP
November 1992	User fee exemption level raised from ZWD 150 to ZWD 400
January 1993	Temporary abolition of fees at rural health centres because of the drought
June 1993	Reinstitution of user fees at rural health centres
January 1994	Substantial increase in user fees at all health institutions
March 1995	Abolition of user fees at rural health centres and rural hospitals
October 1996	Increase in user fees at all referral hospitals: services at rural hospitals and health centres remain free of charge
January 1997	Start of the Health Services fund; retention of user fee revenues at the district and facility level: reinstitution of user fees at (some) rural mission hospitals
1998	No more health grants for the municipalities; higher than average increase in user fees
November 1999	Substantial increase in user fees at government health institutions

Source: Bijlmakers, 2003.

The economic collapse of the early 2000s led to a sharp reduction in public funding for health, and inevitably a shift to private (largely out of pocket) funding. Government health expenditures as a percentage of total health expenditures declined from 36.8% in 1999 to 9.8% in 2005 [22,25]. In the same period, household out-of-pocket spending soared from 23% to 62%. Insurance, which had contributed 20% of health expenditure in 2001 shrank dramatically to 0.9% by 2005, according to National Heath Accounts data. Donors absorbed some of the gap, but given the political problems, this was relatively limited: the share of donor funding increased from 13% to 19% over the period.

Spiralling inflation in the 1990s and the subsequent dollarisation will have affected the affordability of health care, through changes to incomes, prices of health care and the wider cost of living. In 2007, the ‘Access to health services’ study [21] found that 59% of respondents were charged for health care services, especially in urban, large-scale farming and mining areas. Of these, 36% reported inability to pay. No studies to date have examined the overall effect on different segments of the population.

### Current user fees & RMNH services

#### Exemptions

Accounts of which services should be free vary in the written sources. According to one source, the following services and groups are exempt [22]:

•Antenatal care in rural and semi-rural areas

•Referrals to the next highest level of facility for services that the lower-level facility cannot provide

•Directly Observed Treatment Short course (DOTS) for tuberculosis (TB)

•Family planning

•Antiretroviral therapy (ART)

•Emergency outbreak services (such as the recent cholera outbreak)

•Health services for children under five, adults over 65, military veterans, health care providers, and individuals living below the poverty threshold (a designation that is very difficult to attain in practice)

EQUINET states that the policy of free public sector care at rural clinics is still in force and that pregnant women, children under 5 and adults over 65 are exempt from fees up to district level [26]. However, all agree that implementation is patchy and confused. In the recent health system assessment, many providers were unaware of any policy on user fees [22]. Moreover, while the MoHCW has a policy of free care at clinic level, this has not been applied uniformly by local government and mission clinics. Unless lost revenues are replaced through some mechanism, it is unlikely that implementation will be effective, particularly in the non-MoHCW facilities.

The categories reported to be exempt during our fieldwork were under-fives and over-65s, and staff, but with local variations such as the chronically ill (psychiatric patients) and lepers being exempted. Certain donor-supported national programmes are also free (such as ART and TB treatment). It is not surprising that there is variation as there is a perception that charging policies can be locally determined.

There is a Social Dimension Fund Scheme, run by the Ministry of Labour and Social Services, which applies its own criteria for exemption. Orphans, the elderly and the indigent are catered for through this fund. Patients who cannot pay cannot be turned away but can be referred to this fund. However, the money in the Fund is limited and so although the hospital presents claims for people who have been certified and treated for free, they are often not reimbursed as there are no funds.

#### Current levels of fee and ability to pay

Consultation fees for adults, collected from a sample of facilities in 2009, increased according to level of care, with an average of $1 at rural health centres, $3 at mission hospitals, $4 at private health clinics, $4 at district hospitals, $5.5 at provincial hospitals and $9.75 at national hospitals [22].

An official price list is produced by the Association of Health Care Standards of Zimbabwe. However, the prices are high, and facilities produce their own price lists. In the hospital visited, prices for those with Medical Assistance (a voluntary health insurance scheme, of which only a small proportion of their clientele were members) were considerably higher than for those paying cash. The rural clinics used to be free but between 2009 and 2010 they started to charge. Municipal clinics charge more and always have done so.

Prices for a sample of reproductive services are given below, based on the limited qualitative fieldwork undertaken (Table 2).

**Table 2:** Prices quoted for selected RMNH services, research district

**Service**	**Facility type**	**Facility price**
FP – 4 cycles of pills	Government clinics	$0.5
	Municipal & RDC health clinics	$1
FP – Depo Provera injection	Government clinics	$1
	RDC health clinics	$2
	Municipal health clinics	$3
Booking for pregnancy – ANC etc.	Government clinics	$5 for registration, routine monthly examinations and monitoring of weight
	Rural district council facilities	$10 for registration and booking for ANC and $2 for every ANC visit
	Municipal health clinic	$30 total
	Provincial/district hospital	$35 total
Ultrasound	Provincial/district hospital	$25
Normal delivery	Municipal health clinic	$35
Normal delivery	Provincial/district hospital	$50 (but with added items which patients have to buy, this is more likely to come to $100)
Caesarean section	Provincial/district hospital	$450 (simple CS)
		$600 (CS plus observation for six days)
Postnatal check-up	Provincial/district hospital	$10 (then $20 at six weeks)

Source: interviews with informants, research district.

Delivery care in facilities, even in the absence of complications, was not seen as affordable by users or staff. This is likely to be one factor behind the high rate of home deliveries, even though these are discouraged (and traditional birth attendants (TBAs) were nervous about speaking about their work as a result), and despite the fact that families have to bring newborn babies into health facilities to get a birth record.

Our small-scale fieldwork confirms that users are being charged for primary care in rural areas, not least because under the policy of decentralisation the majority of clinics are managed by local authorities, which are able to set their own charging policies. In the area we visited, the rural and urban clinics collect fees but do not retain them. They were reported to receive very little back by way of financial support from district councils and municipalities. They therefore suffer a double disadvantage of higher barriers for users and a lack of funds to reinvest in services.

Many try to avoid paying, so a variety of mechanisms are used to recoup health service costs (ultimately, the public sector at least is not allowed to turn away anyone). Upfront payments are taken for consultation. Patients’ relatives are encouraged to pay, when visiting. People can be allowed to pay in instalments. In some rural areas they pay in kind. Debtors are followed up after discharge. For women who have delivered, the birth record (which is needed for the birth certificate) is withheld until payments are made. In the hospital we visited, staff reported very poor relationships with clients over the issue of non-payment, with women being held in the hospital but in the corridor (as beds were needed) until they paid, or until they absconded.

#### Consequences of fees

The general view based on KII and FGDs with selected health practitioners, user groups and experts was that user fees levied at government-owned health facilities are reasonable compared to those levied by local district council and the municipality. The most punitive aspect of the user fees regime at the council and municipality Family Child Health (FCH) centres, according to mothers who participated in FGDs and nurses in-charge at FCH centres, is the requirement to pay the prescribed fee for every Antenatal Care (ANC) visit. This is borne out by the fact that most mothers forego the routine ANC visits and only present for delivery. Late bookings are another manifestation of failure to afford payment of user fees and cases of booking at seven months were reported. In FGDs mothers reported that late booking was a mechanism to reduce costs that would ordinarily be incurred if one were to book at the recommended first trimester of pregnancy. Factors reported as encouraging home deliveries include long distances to health facilities, people’s inability to afford user fees and fear of being tested for HIV during ANC visits.

Most mothers depend on regular income from formal employment or commercial agriculture to raise money to pay user fees for RMNH services. Not seeking medical attention due to inability to afford user fees is an option that mothers have to take sometimes, to avoid the humiliation that they meet if they present to health facilities without the required fees. Municipality FCH centres are strict and they do not entertain anyone without the requisite fees. The nurse in-charge at the municipality FCH acknowledged that as a result of the strict requirement for payment upfront at municipality clinics there are cases of home deliveries in urban areas.

Home deliveries are common and more openly acknowledged in the rural, small scale and large scale commercial farming sectors than in the urban sector. Those who cannot afford the user fees at the FCH centre seek the help of TBAs for deliveries and are charged a fee of $5 and one bar of laundry soap per delivery. During FGDs with TBAs it emerged that even this lesser charge levied by TBAs was not always readily paid by mothers who were assisted during delivery. The TBAs observed that there were people who simply did not want to pay because they hold the view that TBAs are a community resource and hence should assist their neighbours for no charge.

#### Staff and official perspectives on user fees

From the staff perspective, user fees were a necessary evil as non-payment would in the first instance promote wasteful utilisation of health services and eventually lead to the collapse of the system, given the current low levels of government funding reaching them. There is no direct benefit for staff – it is not legal for staff to receive any of the funds – but indirectly, they benefit through maintenance of services. Besides, some commented that ‘people should make a contribution’.

However, staff in municipal facilities felt that the rates were too high for deliveries, and were putting women off coming there. They considered that they should be reduced, not least because many of the funded public health programmes such as Prevention of Mother to Child Transmission (PMTCT) rely on getting a high throughput of pregnant women in facilities. Others commented that staff should be involved in the setting of fee rates. Post Natal Care (PNC), for example, is charged at a flat rate of $20 at the district hospital, when the supplies involved are minimal (one pair of gloves).

The MoHCW has been carrying out a review of user fees. Given the financial constraints facing the sector, there are concerns about the feasibility of reducing financial barriers for users. However, the inclusive government recognises that user fees are limiting mothers and children’s access to life-saving interventions and advocates for it to be scrapped [27].

## Scope for responding to increased demand for services from a human resource perspective

Changes to access to and utilisation of services have important implications for the health staff in Zimbabwe - if the user fee policy is revised to reduce or remove fees, which would translate into improved access and increased utilization of services, which would in turn increase pressure on staff. In this section, we consider the evidence relating to current stocks of health workers, any gaps in skills, and their distribution. Evidence on current workload is also assessed to establish whether there is scope for increasing the workload of existing staff. We also examine remuneration, which is an important factor behind retention and motivation of staff, and consider what would be the implication of increasing access to RMNH, in terms of the need for and cost of the additional staff.

### Numbers and skills gaps for health workers: recent evidence

The health sector in Zimbabwe has been threatened by the exodus of critical medical skills through migration to countries like South Africa, Botswana, United Kingdom, and New Zealand [28]. It is estimated that more than 80% of the doctors, nurses, pharmacists, radiologists and therapists who trained since 1980 have left the country [29]. This has contributed to declining use of public facilities. The shortage of doctors in Zimbabwe’s public health institutions has also meant heavy workloads for those medical practitioners remaining, particularly those at district hospitals [30,31].

An assessment of maternal and neonatal health services conducted in 2004 found that there were serious shortages of nurses and midwives at the primary levels of care: 40% of primary facilities had no nurse and 50% no midwife at post [32]. In order to manage complications beyond the midwives’ capacity, a standard presence of two Government Medical Officers (GMO) is required at secondary level health facilities. However, 20% of institutions at this level had no GMO at the time of the survey, and 30% had only one in post: patients presenting at these institutions needed to be referred to a higher level of care, thus increasing delays in receiving care. Furthermore, over 30% of secondary facilities did not have a Nurse Anaesthetist at post at the time, which precluded offering caesarean sections in those institutions. Shortages of laboratory technicians were also noted, and of specialists, such as paediatricians and obstetricians, at tertiary levels.

The economic depression and hyperinflation experienced in the mid-2000s heavily affected the stability of health workers in the public sector. The Zimbabwe HRH profile [33] reports that at the peak of the economic depression in 2008, the MoHCW lost 3,588 staff through resignations. The capacity to produce health professionals in Zimbabwe was heavily impacted by the economic depression. Health training schools lost many of their teaching staff through international migration. Professional migration led to the closure of some schools in 2008.

The numbers of health workers have been increasing in 2007–9, with the overall ratio of health workers to population reaching 2.25 per 1,000 population by 2009 [34]. This is just below the overall WHO norm. Nurses (a category including midwives) reached 1.34 per 1,000. Nurses and midwives constitute the largest group within the Zimbabwe health workforce – 46% and 19% of the total respectively, while doctors constituted only 7% in 2009 [34]. The Gupta and Dal Poz [34] study also found that around 90% of the health workforce worked in government-operated facilities.

The average vacancy rate for critical cadres (such as doctors) over 2005–9 was 50%, although for most groups the vacancy rates dropped over the period [34]. It stood at 20% overall for 2010 – not assisted by a hiring freeze which was introduced that year. For nurses, vacancies fell from 87% in 2005 to 44% in 2006, 28% in 2007 and 2008, 14% in 2009, and 10% in 2010 [35]. Attrition rates also dropped over the period. However for some categories the vacancies remained very high; for trainee midwives, for example, there were 97% vacancies in 2010 – which was actually an improvement on 2006, 2008 and 2009 when it was 100% (and 49% in 2007) [35]. Of the 8 posts for Provincial Maternal Child Health (MCH) Medical Officers, two posts were filled in 2010, while for the Reproductive Health Programme, which was supposed to have three top staff, only one (the director) was in post. Key informants also highlight the lack of specialist staff – not one obstetrician/gynaecologist is listed in the posts for 2010, for instance (not even at central hospital level). In addition, the MoHCW and Health Services Board (HSB), which was established by the Health Service Act (15:16) of 2004 [36], to administer the appointment and conditions of service of HRH in the public sector, emphasise the need for the establishment posts to be reviewed, in light of recent changes in needs and workload [37].

Although there are plans to re-attract health staff in the southern African diaspora, most key informants thought it too early for this to be effective, given the general conditions in Zimbabwe. However, some foreign medical staff continued to be recruited, including from Cuba and the Democratic Republic of Congo [35].

### Distribution of staff

As well as gaps in established posts, there are also disparities between posts in rural and urban areas, and proportions of posts filled. Within urban areas, Harare and Bulawayo absorb a large proportion of skilled staff [38]. Mission facilities are also reported to have lower vacancy levels, presumably due to their reportedly higher levels of incentive payments [22].

We analysed data on human resource distribution, drawn from the regular regional returns to the MoHCW and only including the public sector. District data were not available and the data did not separate midwives from other nursing staff. In 2010, there were 17,756 nurses, midwives, clinical officers and doctors working in the public health sector (Table 3). Outside Bulawayo and Harare, nurses appeared to be reasonably evenly spread relative to population, varying from 95 per 100,000 in Manicaland to 151 in Matabeleland South.

**Table 3:** Distribution of medical staff by region (2010)

**Region**	**Doctors**		**Clinical officers**		**Nurses**		**Skilled health workers**	
	**Number**	**Per 100,000**	**Number**	**Per 100,000**	**Number**	**Per 100,000**	**Number**	**Per 100,000**
Bulawayo	168	29.57	-	-	2,460	432.98	Z628	462.55
Harare	349	23.92	20	1.37	4,309	295.38	4,678	320.67
Manicaland	26	1.68	3	0.19	1,476	95.60	1,505	97.48
Mashonaland Central	16	1.61	4	0.40	1,160	116.53	1,180	118.54
Mashonaland East	24	2.17	3	0.27	1,215	110.08	1,242	112.53
Mashonaland West	29	2.48	2	0.17	1,184	101.31	1,215	103.96
Masvingo	17	1.29	1	0.08	1,606	121.72	1,624	123.08
Matabeleland North	15	2.23	-	-	947	140.62	962	142.85
Matabeleland South	22	3.37	-	-	991	151.75	1,013	155.12
Midlands	23	1.97	2	0.17	1,684	143.89	1,709	146.62
TOTAL	689	6.47	35	0.33	17,032	159.85	17,756	166.64

**Figure 1:**
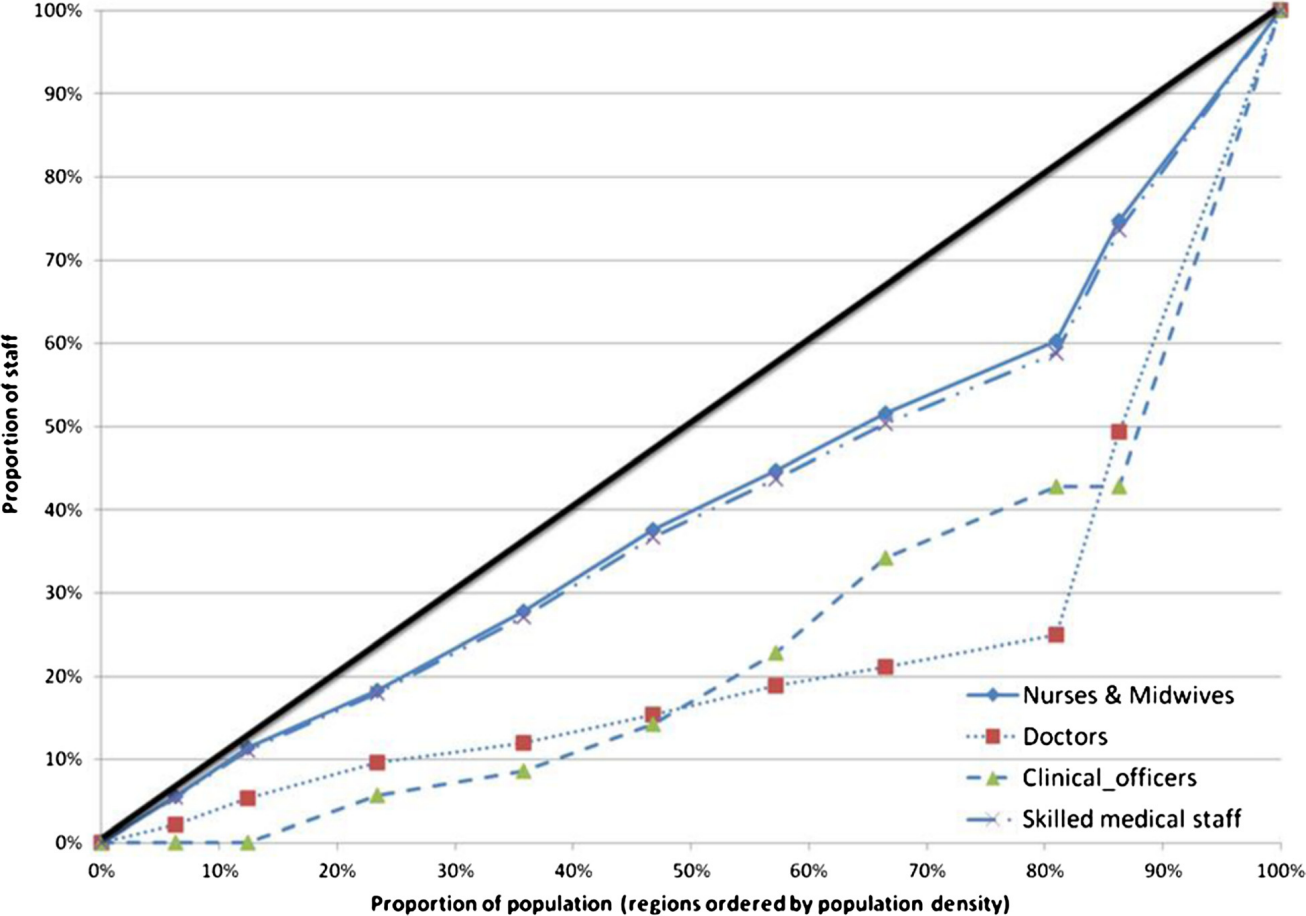
Concentration curve for medical staff (ordered by population density per region).

Doctors were unevenly spread throughout the country. A concentration curve, and accompanying concentration index, for medical staff ordered by population density across the regions shows a strong pro-urban bias for doctors (0.53) and clinical officers (0.52) (Figure 1). For nurses and midwives the distribution was much more even (the concentration index indicates that the distribution is not significantly different from proportionality/equality).

### Remuneration of health workers

HRH expenditure fell from 2005 through 2007, with a complete collapse in human resource spending in 2008, when human resources spending accounted for 0.3% of the public health budget [22]. Realizing that the health sector was in crisis, the MoHCW and partners developed an Emergency Retention Scheme in 2008 to cover the whole public sector including council employees in rural and urban areas. The scheme involved topping up government salaries with retention payments. In March 2009, the government and its partners revised the retention scheme to only apply to grades C5 and above of the MoHCW posts. There seems to be evidence suggesting that the retention scheme, currently managed by Crown Agents, has brought some stability in the public health sector. The number of resignations in 2009 dropped to 84% (567) of the previous year [34], although this may also be affected by the dollarisation introduced in 2009.

The top-up payments, funded by partners, were higher than base salaries for some grades. For example, a District Nursing Officer in 2008 received $250 as monthly salary and $280 as retention payment. The differential for higher grades was even greater. Moreover, mission facilities and municipal facilities, while they share the same base salaries as the MoHCW facilities, are able to provide higher retention payments and allowances, leading to a new form of internal migration. The current City Health Council salaries and benefits are reported to be three times the MoHCW ones (inclusive of the retention allowance) [34]. In general, dollarisation has brought stability to incomes and prices but may also have increased the cost of living. The effects on health workers have yet to be studied. Comparisons of wages across the sectors are also not available on a systematic basis.

According to the HSB Annual Report for 2010 [35], salaries for General Hands (support staff) were $127 per month, while State Registered Nurses received $176 and Junior Doctors $218. Meanwhile, the total consumption poverty line for a family of average size (5 people) was estimated at $533 in July 2011 [39]. In addition to base salary, various allowances are paid as follows:

•Uniform allowance: $15 per month

•Residence: $150 per month

•Stand-by/on call: 20% of salary

•Transport allowance: minimum of $8 per month.

Some loans are also available to help with housing and car purchase – however, the sums available were reported to be well below needs.

The overall level of remuneration is therefore very low, and the retention payments (currently funded by the Global Fund and only available to staff above C5 level) are being reduced by 25% each year, with the aim of phasing out in 2013. There are deep concerns about the effects which this may have on retention (see Table 4 for summary of overall challenges, as presented by key informants).

**Table 4:** Summary of HRH challenges, based on key informant interviews

	
1	The HR establishment is not matched to its task – programmes and populations have grown but the establishment has not been adjusted accordingly. The staffing norms have not been adjusted since the 1980s and the MoHCW and HSB recognise that this is overdue. They are planning to revise using the WHO workload model, but it is hard to justify this exercise when existing positions remain vacant.
2	In addition, there has been a hiring freeze since mid 2010, so even the existing posts, if vacant, cannot be filled (except with permission from the Ministry of Finance, which takes 6–7 months to obtain) and it is difficult to transfer staff.
3	The level of salaries is universally acknowledged to be too low – below the consumption poverty line for an average family.
4	Differentials between sectors add to difficulties for government facilities – a qualified midwife earns $300 in the public sector (up to $400 including all allowances), but can get $1,000 per month in Harare city facilities, according to one key informant.
5	The retention allowance is also low - $70 per nurse – and is sometimes delayed. In addition, it is not paid to the non-professional grades, which is demotivating. The allowance, currently funded by the Global Fund, is also reducing by 25% each year, and is due to phase out in 2013.
6	There is a shortage of specialists, including doctors, midwives and specialist nurses. 60% of nurses should have qualifications in midwifery, according to one key informant, but the actual level is far below that. The provincial hospital visited, to cite one example, has no paediatrician, no obstetrician, and only one doctor and one surgeon. The last time they had a Zimbabwean specialist, according to the key informant, was over 20 years ago (they have hosted Cuban doctors, but these present language problems).
7	Migration, while reduced compared to the ‘rock bottom years’ of the mid-2000s, continues to drain trained staff, especially to South Africa and Botswana.
8	Maldistribution is also a recognised problem, reflecting poorer working conditions and earning opportunities. A rural allowance used to exist but was considered too low to be effective (25% of a small salary).
9	As a consequence of these factors, remaining staff are often overloaded, which contributes to demotivation.
10	Poor personal and working conditions are also mentioned by many staff – for example, lack of staff accommodation, lack of transport to work, dirty wards, lack of staff amenities, and no running water.
11	Shortages of key supplies (such as blood) and equipment at work also undermines their professional self-respect and ability to offer a reasonable quality of care.
12	The lack of specialists denies remaining staff the opportunity to learn and improve their skills, while trainees mention the absence of senior staff to supervise them.
13	A result-based management system exists in theory, based on annual targets and appraisals, but the system is seen as cumbersome and the increments to reward good performance are too minimal to motivate.

Dollarisation and other recent changes have improved the situation of the health workforce (who at the lowest point in 2008/9 were paid $1 per month). However, expectations of a continuous improvement have not been met. Looking ahead, there is concern about ongoing economic and political problems and fears that things may even get worse – if, for example, the health workforce is ‘streamlined’.

Table 5 shows the pay scales for 3 cadres of health workers in US$, and expressed as a ratio of salary to GDP per capita^b^.

**Table 5:** Monthly salaries expressed in US dollars and ratio of salary: GDP per capita

**Cadre**	**US$/month**	**Ratio of salary: GDP per capita**
General hand (support staff)	127	2.56
Registered nurse	176	3.55
Doctor	218	4.40

Source: HSB Annual Report (2010).

Health workers are paid above the much depleted average income level but not to the same extent as in other countries, and it is likely that the value of pay has fallen considerably since before the crisis. Doctors’ salaries are about 4-fold average GDP per capita.

### Workload

There were around 12 deliveries for each skilled health worker per year and 313 for each doctor across the country in 2010 (Table 6). If all births were to be conducted with assistance from a skilled health worker this number would increase to 18 per health worker or around 475 per doctor. WHO suggests that 1 doctor is required for around 1,000 births, to provide emergency intervention where there are complications before, during and after delivery, while a midwife can provide care for 175 births per year. According to the most recent Demographic Health Survey (DHS), around two thirds of deliveries are undertaken in facilities with a skilled health worker [40]. Masvingo, Midlands, Manicaland and Mashonaland Central currently have fewer doctors than required to provide care for the current workload in facilities. Across the country, there appear to be sufficient doctors to provide care for all births but the distribution means that only in Bulawayo, Harare and Matabeleland South are there sufficient doctors to provide cover for all births in the event of complications. It is more difficult to evaluate provision of skilled birth attendants. The human resource statistics do not distinguish between midwives and other nursing staff. If all nurses are regarded as skilled birth attendants there are around 18 births per skilled health worker, ranging from 4.3 in Bulawayo to 32.6 in Manicaland. This suggests that there is ample capacity to provide for the existing number of deliveries and indeed to permit a scale-up. Yet this conclusion is too simplistic since whether the existing staff is sufficient to provide services to all deliveries depends on a number of factors including:

•how much of their time they devote to non-reproductive health services

•their distribution within each region

•the extent to which the skills of nurses currently qualify them to provide adequate delivery health services.

Since most nurses lack midwifery skills it is likely that the number of medical workers considerably over -estimates the capacity to provide midwifery care to an adequate standard.

**Table 6:** Annual delivery workload relative to population and skilled staff (2010)

**Region**	**Deliveries**			**Births**		
	**/100,000 population**	**/skilled health worker**	**/Doctor**	**/100000 population**	**/skilled health worker**	**/Doctor**
Bulawayo	1,785.68	3.86	60.39	2,020.00	4.37	68.31
Harare	2,201.89	6.87	92.04	2,637.00	8.22	110.23
Manicaland	1,916.59	19.66	1,138.10	3,178.42	32.61	1,887.39
Mashonaland Central	1,665.45	14.05	1,036.15	3,240.18	27.33	2,015.85
Mashonaland East	1,788.83	15.90	822.66	2,986.36	26.54	1,373.39
Mashonaland West	1,813.08	17.44	730.67	3,296.51	31.71	1,328.49
Masvingo	2,239.04	18.19	1,737.81	2,977.45	24.19	2,310.92
Matabeleland North	1,972.13	13.81	885.40	3,001.73	21.01	1,347.63
Matabeleland South	2,094.49	13.50	621.73	2,925.26	18.86	868.34
Midlands	2,553.20	17.49	1,299.22	3,946.22	27.02	2,008.06
Total	2,024.89	12.15	313.14	3,074.12	18.45	475.40

### Projected need for RMNH workforce if demand is increased

The MPS tool, produced by WHO, provides a simple and graphical analysis of the quantity and cost of skilled birth attendants and doctors as coverage is scaled up^c^. Using 2010 as the baseline year, we have estimated the number of additional staff needed to reach near universal coverage (95%) by 2015.

The model is quantity driven. It assumes that the current level of staff is just sufficient to provide the current level of skilled deliveries. Any increase in coverage requires additional skilled birth attendants (SBAs) and doctors based on standard ratios: around 1,000 deliveries per doctor and (a user adjustable) 175 deliveries per SBA. The model permits users to specify levels of attrition for staff and salaries and assumed salary increases to permit cost projections. The number of births is based on demographic projections for the country. Midpoint annual salaries (2010) for registered nurses and doctors as computed above are used for the projection of recurrent cost and these are expected to increase by 3% per annum (in dollar terms) (see Table 7). An initial level of coverage of 66% of deliveries with a skilled health worker is assumed which corresponds to levels reported in the provisional DHS for 2010 and is consistent with the HMIS data analysed above [40].

**Table 7:** Summary assumptions for Zimbabwe staffing needs projections

	**Deliveries per year**	**Baseline salary (US $)**	**Growth in salaries**	**Annual attrition**
**SBA**	175	2112	3%	10%
**Dr.**	1000	2616	3%	10%

The presumption that coverage can be increased by adding staff in a fixed ratio to deliveries is an important one. It assumes that the main constraint to increasing attended deliveries is one of supply. Yet it is clear from the analysis of actual data that in some areas high levels of attended deliveries are possible with a relatively small staffing whilst in other areas a high level of staffing is associated with a modest level of attended deliveries. Various factors may account for these differences including that:

•Staff may be busy with other activities and only able to devote a small proportion of time to delivery care. This is dealt with by focusing on full time equivalents at least for the additional staff required.

•Staff may not be motivated to provide services

•Staff have insufficient resources to provide adequate care.

•Human resources are available at a facility but women are impeded from seeking care due to inaccessibility or high cost of transport and lack of knowledge about when to seek care. Both these factors are known to be associated with levels of delivery.

These factors could mean that a much higher ratio of staff to deliveries may be required in order to achieve universal access or indeed that targets are unattainable unless other barriers to use of services are addressed. Conversely, it may be that current staffing levels are sufficient to deal with more deliveries but that other factors prevent scale up.

The projections, based on regional data and factoring in attrition of staff, suggest that a scale up from 66% to 95% by 2015 will not require additional staff at national level, and even if we are correct about 20% of those staff we have assumed have midwifery skills, this remains the case. A total of 1,778 skilled birth attendants or midwives and 311 doctors are required to provide for 95% coverage. There are currently 17,032 nurses and 689 doctors in the country.

**Figure 2:**
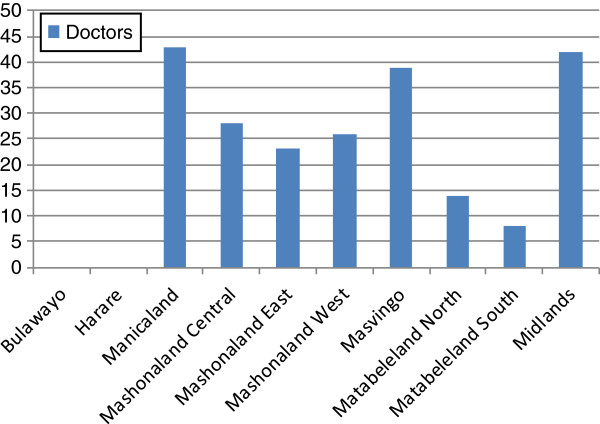
Gaps in numbers of doctors required by region.

However, given the current distribution of the available staff, across the country the requirements for additional staff vary (Figure 2). In regions other than Bulawayo and Harare, scale-up suggests the need for additional health workers. In Manicaland, for example, scale-up from current coverage of around 60% to 95% is estimated to require a further 43 doctors from a current estimated need of 29 (statistics show there are currently 26 doctors in the region). Figure 2 identifies the shortfall as 223 doctors required outside Harare and Bulawayo. The relatively high ratio of other staff classed as skilled attendants to births, and the relatively even distribution of other staff around the country ensure that there are sufficient skilled attendants other than doctors in all regions. At worst (in Manicaland and Mashonaland West), this would still apply if only 30% of those staff do indeed possess sufficient midwifery skills.

The model produces the odd result that the cost of the health workforce declines from 2010 to 2015. This is because it treats staffing as more than adequate for RMNH purposes and allows attrition at 10%, faster than salary growth at 3%, only replacing staff lost to the system when numbers fall below those required to deliver the target assisted delivery rate. The current cost of the workforce is estimated at $37.7m, falling to $25.8m in 2015. These figures are also influenced by the relatively low dollar salaries of medical workers and the low compression ratio between the salaries of doctors and other medical workers.

## Discussion and conclusions

Zimbabwe has been hit by an economic crisis over the past decade or so, causing worrying deterioration in reproductive health indicators (a sizeable increase in maternal mortality, reduction in facility deliveries, and a substantial rise in neonatal mortality, though this has improved in recent years). Public investment in health care is low, and poverty is widespread.

The past decades have seen a shift in the burden of payments onto households. Implementation of the complex rules on exemptions is patchy and confused. In addition, non-MoHCW facilities (which include municipal and mission facilities) have complete discretion on charging. Most staff and women see delivery care in facilities as hard for families to afford, even in the absence of complications. Moreover the fees generated are not retained in municipal and rural district-run facilities, thus creating a vicious cycle of low utilisation and low investment in the services. Central funding of non-salary recurrent costs, especially for district services, appears to be very low.

Staff numbers have been reduced through emigration to other countries and also internal migration to other sectors, though this is less well documented. There is a particular shortage of midwives and of specialists, though for most groups in the past few years numbers have been increasing, while attrition rates have reduced. There are however discrepancies in vacancies across regions and sectors.

Our analysis suggests that nurses and midwives are evenly distributed across the country (at least in the public sector, for which data is available), though doctors are not. This means that for some provinces (Masvingo, Midlands, Manicaland and Mashonaland Central), there are not enough doctors to provide more complex care, and only three provinces could provide cover in the event of all deliveries taking place in facilities. For midwives and nurses, there appear to be adequate numbers but the merger of categories means that assessing competence in obstetric care is hard and there are likely to be skills shortages for existing staff.

Pay is recognised to be low – below the poverty line for an average family. There are also concerns about the effects of the ending of the emergency retention payments, which are due to be phased out shortly. Health workers are paid above the much depleted average income level but not to the same extent as in other countries, and it is likely that the value of pay has fallen considerably since before the crisis. Doctors’ salaries are about 4-fold average GDP per capita.

This analysis suggests that there are a number of axes of challenge in Zimbabwe, all of which hinge on improved economic fortunes. There is a strong case for reducing the financial burden on clients of RMNH services and also a pressing need to improve the terms and conditions of key health staff. Numbers need to grow, and distribution is also a challenge, suggesting the need for differentiated policies in relation to rural areas, especially for doctors and specialists. The management of user fees should also be reviewed, particularly for non-MoHCW facilities which do not retain their revenues, and appear to receive limited investment in return from the municipalities and district councils. Overall public investment in the health system (including increased external support, if the political situation permits it) needs to grow.

## Endnotes

^a^The CPI index for health went from 100 in 2001 to 214 in 2002, 775 in 2003, 4,224 in 2005, 18,151 in 2005, and 595,497 in 2006, according to ZIMSTATS Quarterly Digest, July 2011. Later figures are not yet published.

^b^Accessed 15th December 2011. Appropriate purchasing power parity (PPP) conversion factors that would allow for the estimation in international dollars are not available for Zimbabwe. The most recent PPP data are for 2009 and convert Zimbabwe $. They do not therefore relate to the current use of US$. However, it cannot be assumed that the purchasing power of 1US$ is the same in Zimbabwe and the US.

^c^The original model (http://mps.projection.free.fr/mdg5-hrsu.html) is available as an online and offline web-based. For the purposes of this study, the model was converted to Excel and adapted to permit variations in the base and target years and differences in attrition rates and salaries for doctors and nurses/midwives.

## Competing interests

The authors declare that they have no competing interests.

## Authors’ contributions

YC led the research group, including participation in data collection, analysis and drafting of final article. SW led on overall study design, participated in data collection and led on analysis and drafting. MM participated in data gathering and analysis, focussing on secondary data. WM participated in data gathering and analysis, focussing on fieldwork. TE participated in study design and led on analysis of workload and need for HRH data. BM led on overall international study design and analysis of remuneration data. SM led the Zimbabwe team and contributed to study design. All authors have approved the final version of the article.

## Pre-publication history

The pre-publication history for this paper can be accessed here:

http://www.biomedcentral.com/1472-6963/13/197/prepub
